# Exposure to Electronic Cigarette Advertising Among Middle and High School Students — United States, 2014–2016

**DOI:** 10.15585/mmwr.mm6710a3

**Published:** 2018-03-16

**Authors:** Kristy Marynak, Andrea Gentzke, Teresa W. Wang, Linda Neff, Brian A. King

**Affiliations:** 1 Office on Smoking and Health, National Center for Chronic Disease Prevention and Health Promotion, CDC.

Electronic cigarettes (e-cigarettes) are the most commonly used tobacco product among U.S. middle and high school students (*1*). Exposure to e-cigarette advertisements is associated with higher odds of current e-cigarette use among middle and high school students (*2*–*4*). To assess patterns of self-reported exposure to four e-cigarette advertising sources (retail stores, the Internet, television, and newspapers and magazines), CDC analyzed data from the 2014, 2015, and 2016 National Youth Tobacco Surveys (NYTSs). Overall, exposure to e-cigarette advertising from at least one source increased each year during 2014–2016 (2014: 68.9%, 18.3 million; 2015: 73.0%, 19.2 million; 2016: 78.2%, 20.5 million). In 2016, exposure was highest for retail stores (68.0%), followed by the Internet (40.6%), television (37.7%), and newspapers and magazines (23.9%). During 2014–2016, youth exposure to e-cigarette advertising increased for retail stores (54.8% to 68.0%), decreased for newspapers and magazines (30.4% to 23.9%), and did not significantly change for the Internet or television. A comprehensive strategy to prevent and reduce youth use of e-cigarettes and other tobacco products includes efforts to reduce youth exposure to e-cigarette advertising from a range of sources, including retail stores, television, the Internet, and print media such as newspapers and magazines (*5*).

Data were analyzed from the 2014, 2015, and 2016 NYTSs, a cross-sectional, paper-and-pencil survey administered to U.S. students in grades 6–12.* NYTS utilizes a three-stage cluster sampling design to generate a nationally representative sample of public and private school students. Sample sizes and response rates for 2014, 2015, and 2016 were 22,007 (73.3%), 17,711 (63.4%), and 20,675 (71.6%), respectively.

Participants were asked “how often do you see advertisements or promotions for electronic cigarettes or e-cigarettes” from the following four sources: 1) “when you are using the Internet”; 2) “when you read newspapers or magazines”; 3) “when you go to a convenience store, supermarket, or gas station”; and 4) “when you watch television or go to the movies.” Movies were omitted from the question after 2014. Response options for each question were “I do not [use/visit the source]”; “never”; “rarely”; “sometimes”; “most of the time”; and “always.” Consistent with previous research, students who reported “sometimes,” “most of the time,” or “always” were classified as “exposed” to advertisements from each source; those who selected “never,” “rarely,” or “I do not [use/visit the source]” were classified as “not exposed” (*6*). The number of exposure sources were summed for each student and reported as the percentage of all students who were exposed to one, two, three, or four sources.

Data were weighted to account for the complex survey design and adjusted for nonresponse. Prevalence estimates and 95% confidence intervals of exposure to each source, and to any source, were computed. Estimates of exposure were assessed overall and by sex, race/ethnicity, school grade, current (past 30-day) use of e-cigarettes, and current (past 30-day) use of any other tobacco product.^†^ Within each year, t-tests were used to assess statistically significant differences between levels of each covariate relative to the referent group (p<0.05). Between-year differences in the overall percentage of students exposed to each advertisement source during 2014–2016 were assessed using the Wald F test and posthoc corrections for multiple hypothesis testing (p<0.0167).^§^

Among U.S. middle and high school students during 2014–2016, exposure to e-cigarette advertisements from any source increased from 68.9% (18.3 million) to 78.2% (20.5 million) (Figure 1) (Table). In 2016, exposure was highest for retail stores (68.0%, 17.7 million), followed by the Internet (40.6%, 10.6 million), television (37.7%, 9.7 million), and newspapers and magazines (23.9%, 6.2 million). In 2016, exposure to advertising from any source was more prevalent among females (79.9%) than males (76.5%); non-Hispanic whites (79.6%) than Hispanics (77.0%) and students of other non-Hispanic races/ethnicities (73.6%); 8th (78.5%), 10th (81.0%), 11th (79.3%), and 12th graders (79.0%) than 6th graders (75.0%); high school students (79.2%) than middle school students (76.9%); current e-cigarette users (82.8%) than nonusers (77.9%); and current users of other tobacco products (82.7%) than nonusers (77.6%). Exposure to each advertising source was higher among current e-cigarette users and other tobacco product users than nonusers during 2014, 2015, and 2016 (Table).

**FIGURE 1:**
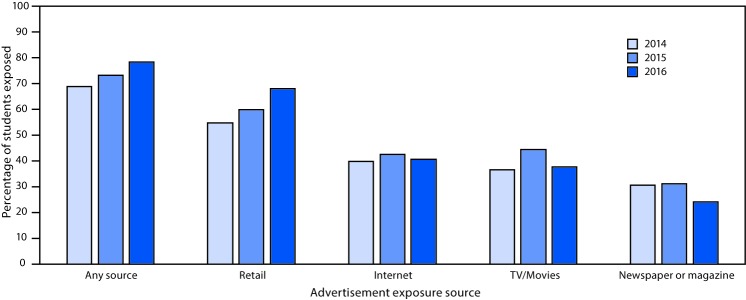
Percentage* of U.S. middle and high school students exposed to e-cigarette advertisements through any source,^†^ retail stores,^§^ the Internet,^¶^ television/movies,** and newspapers and magazines^††^ — National Youth Tobacco Survey, United States, 2014–2016 * Between-year differences in the percentage of students exposed to each advertisement source during 2014–2016 were assessed using the Wald F test and posthoc corrections for multiple hypothesis testing (p<0.0167). ^†^ Statistically significant increases occurred during 2014–2015, 2015–2016, and 2014–2016. ^§^ Statistically significant increases occurred during 2014–2015, 2015–2016, and 2014–2016. ^¶^ Statistically significant increase occurred during 2014–2015. ** Statistically significant increase occurred during 2014–2015; statistically significant decrease occurred during 2015–2016. Movies were removed as an exposure source after 2014. ^††^ Statistically significant decreases occurred during 2015–2016 and 2014–2016.

**TABLE:** Prevalence of exposure to e-cigarette advertisements* among U.S. youths by sex, race/ethnicity, school level, and use of e-cigarettes and other tobacco products by exposure source — National Youth Tobacco Survey, United States, 2014–2016

Demographic characteristic/Year	% (95% CI)				
	Retail stores	Internet	Television /Movies	Newspapers and magazines	Any source
**Overall**					
2014	54.8 (53.6–56.0)	39.8 (38.5–41.1)	36.5 (35.3–37.7)	30.4 (29.3–31.6)	68.9 (67.7–70.0)
2015	59.9 (58.2–61.7)	42.6 (40.8–44.4)	44.5 (42.7–46.2)	31.0 (29.9–32.2)	73.0 (71.3–74.5)
2016	68.0 (66.9–69.1)	40.6 (39.5–41.8)	37.7 (36.1–39.3)	23.9 (22.9–24.9)	78.2 (77.1–79.1)
**Overall population estimate (in millions)^†^**					
2014	14.4	10.5	9.6	8.0	18.3
2015	15.7	11.1	11.6	8.1	19.2
2016	17.7	10.6	9.7	6.2	20.5
**Sex**					
Male (referent)					
2014	54.6 (52.9–56.4)	38.5 (37.1–39.8)	36.7 (35.2–38.2)	28.7 (27.6–29.9)	69.0 (67.6–70.3)
2015	58.1 (56.1–60.0)	39.4 (37.6–41.3)	42.9 (40.9–45.0)	28.3 (27.0–29.7)	71.3 (69.3–73.1)
2016	66.3 (64.9–67.7)	37.5 (36.3–38.7)	34.8 (33.2–36.5)	21.8 (20.6–22.9)	76.5 (75.2–77.7)
Female					
2014	54.9 (53.5–56.3)	41.1 (39.4–42.9)^§^	36.4 (34.8–38.0)	32.1 (30.2–34.1)^§^	68.8 (67.3–70.3)
2015	62.1 (60.1–64.0)^§^	46.0 (43.8–48.2)^§^	46.0 (44.3–47.9)^§^	33.8 (32.2–35.4)^§^	74.9 (73.0–76.6)^§^
2016	69.8 (68.3–71.1)^§^	43.7 (42.2–45.3)^§^	40.5 (38.5–42.5)^§^	26.0 (24.7–27.3)^§^	79.9 (78.7–81.0)^§^
**Race/Ethnicity**					
White, non-Hispanic (referent)					
2014	56.7 (55.0–58.4)	40.2 (38.5–42.0)	35.2 (33.7–36.6)	31.1 (29.7–32.5)	70.4 (68.8–72.0)
2015	63.8 (61.3–66.2)	44.2 (41.8–46.6)	46.0 (43.5–48.4)	33.1 (31.7–34.6)	75.3 (73.2–77.2)
2016	71.3 (69.9–72.8)	41.0 (39.3–42.6)	36.2 (34.1–38.4)	25.1 (23.6–26.6)	79.6 (78.3–80.8)
Black, non-Hispanic					
2014	51.7 (49.4–53.9)^¶^	41.3 (38.5–44.2)	42.2 (40.0–44.3)^¶^	32.2 (30.0–34.5)	68.6 (66.3–70.8)
2015	56.7 (54.2–59.1)^¶^	41.8 (39.2–44.6)	47.1 (44.9–49.3)	27.9 (25.6–30.3)^¶^	72.8 (70.6–75.0)^¶^
2016	63.6 (61.5–65.7)^¶^	39.7 (37.3–42.2)	43.8 (41.3–46.3)^¶^	21.0 (19.4–22.7)^¶^	78.5 (76.4–80.5)
Hispanic					
2014	55.6 (53.8–57.4)	39.4 (37.8–41.1)	37.4 (35.6–39.4)^¶^	29.2 (27.1–31.3)	68.9 (67.2–70.6)
2015	55.8 (53.7–57.9)^¶^	40.4 (38.3–42.6)^¶^	42.2 (40.1–44.3)^¶^	29.4 (27.8–31.1)^¶^	70.5 (68.4–72.6)^¶^
2016	65.9 (64.4–67.5)^¶^	41.9 (40.2–43.6)	39.1 (37.1–41.2)^¶^	23.4 (22.0–24.9)	77.0 (75.3–78.6)^¶^
Other, non-Hispanic					
2014	44.4 (39.2–49.7)^¶^	32.6 (28.3–37.2)^¶^	29.9 (26.1–33.9)^¶^	25.3 (22.1–28.7)^¶^	58.3 (52.4–63.9)^¶^
2015	51.1 (47.5–54.7)^¶^	39.3 (35.1–43.6)^¶^	35.6 (32.8–38.5)^¶^	26.6 (23.3–30.2)^¶^	63.8 (59.7–67.6)^¶^
2016	62.6 (58.6–66.4)^¶^	37.0 (33.5–40.6)	31.9 (27.5–36.6)	22.9 (20.1–25.8)	73.6 (70.0–76.9)^¶^
**Grade level**					
6th grade (referent)					
2014	50.6 (47.2–54.0)	32.8 (30.8–34.8)	31.8 (29.4–34.3)	24.1 (22.1–26.2)	64.7 (61.9–67.3)
2015	52.7 (49.2–56.2)	35.5 (31.9–39.4)	40.8 (37.5–44.2)	24.4 (22.1–26.9)	66.7 (62.7–70.4)
2016	62.9 (60.0–65.8)	38.4 (35.4–41.5)	34.4 (31.3–37.5)	17.2 (15.5–19.2)	75.0 (72.4–77.4)
7th grade					
2014	55.0 (51.7–58.3)	36.7 (34.4–39.0)**	35.6 (32.8–38.5)**	25.9 (24.0–28.0)	67.8 (65.1–70.3)
2015	60.3 (57.5–63.1)**	40.3 (37.5–43.1)**	44.2 (41.1–47.4)**	27.4 (24.5–30.4)	72.6 (69.8–75.3)**
2016	66.2 (63.5–68.7)**	41.4 (38.7–44.2)	36.9 (34.0–39.9)	21.0 (19.2–22.9)**	77.3 (75.1–79.4)
8th grade					
2014	52.6 (48.9–56.3)	37.6 (34.7–40.5)**	34.6 (32.2–37.1)**	25.0 (21.5–28.9)	66.6 (63.4–69.6)
2015	59.7 (56.4–63.0)**	41.2 (37.4–45.1)**	43.5 (39.7–47.3)	29.6 (27.1–32.2)**	73.9 (70.7–76.9)**
2016	67.8 (65.1–70.3)**	38.5 (35.8–41.3)	36.6 (33.7–39.7)	22.0 (19.9–24.3)**	78.5 (76.4–80.4)**
9th grade					
2014	54.7 (52.1–57.2)	39.2 (37.0–41.4)**	37.2 (34.9–39.7)**	32.0 (30.1–34.0)**	68.7 (65.9–71.4)
2015	60.4 (57.8–62.8)**	45.4 (42.8–48.0)**	46.6 (44.3–49.0)**	32.2 (30.1–34.3)**	74.8 (72.8–76.7)**
2016	68.0 (65.5–70.5)**	39.5 (37.3–41.8)	37.4 (34.6–40.3)	23.7 (21.9–25.5)**	77.6 (75.4–79.7)
10th grade					
2014	56.2 (53.6–58.8)**	43.4 (40.9–45.8)**	38.9 (36.5–41.3)**	34.0 (31.6–36.5)**	71.3 (68.8–73.7)**
2015	60.2 (57.5–62.8)**	43.8 (40.6–47.0)**	43.7 (41.2–46.3)	32.4 (30.0–34.9)**	72.5 (70.0–74.9)**
2016	71.6 (69.4–73.8)**	44.0 (41.6–46.4)**	39.8 (37.3–42.4)**	27.8 (25.5–30.2)**	81.0 (78.9–82.9)**
11th grade					
2014	57.8 (54.9–60.6)**	45.5 (43.3–47.6)**	39.9 (37.1–42.7)**	35.9 (33.7–38.1)**	71.8 (69.3–74.1)**
2015	63.1 (58.9–67.2)**	45.8 (42.9–48.7)**	45.9 (42.8–49.0)**	35.5 (32.7–38.4)**	74.1 (70.8–77.1)**
2016	69.8 (67.4–72.1)**	41.6 (39.2–44.0)	40.4 (37.4–43.4)**	26.9 (24.6–29.4)**	79.3 (77.3–81.3)**
12th grade					
2014	56.8 (54.2–59.3)**	44.1 (41.7–46.6)**	37.8 (34.5–41.3)**	37.1 (34.7–39.5)**	71.9 (69.6–74.1)**
2015	64.4 (61.2–67.5)**	46.8 (43.3–50.3)**	46.8 (44.3–49.3)**	36.9 (34.8–39.1)**	77.0 (74.4–79.4)**
2016	70.8 (67.9–73.5)**	41.3 (38.3–44.2)	38.7 (35.3–42.2)	29.6 (27.7–31.6)	79.0 (76.5–81.3)**
**School level**					
Middle school (referent)					
2014	52.8 (50.9–54.7)	35.8 (34.2–37.4)	34.1 (32.3–35.8)	25.0 (23.8–26.3)	66.4 (64.9–67.9)
2015	57.6 (55.1–60.1)	39.0 (36.3–41.8)	42.8 (40.0–45.7)	27.1 (25.5–28.9)	71.1 (68.4–73.6)
2016	65.6 (63.9–67.3)	39.5 (37.7–41.3)	36.0 (33.9–38.1)	20.1 (18.9–21.4)	76.9 (75.2–78.5)
High school					
2014	56.3 (54.7–57.9)^††^	42.9 (41.4–44.4)^††^	38.4 (36.8–40.1)^††^	34.6 (33.3–36.0)^††^	70.9 (69.3–72.4)^††^
2015	61.9 (60.1–63.7)^††^	45.4 (43.8–47.0)^††^	45.7 (44.2–47.3)^††^	34.1 (32.9–35.4)^††^	74.5 (73.1–75.9)^††^
2016	70.0 (68.4–71.6)^††^	41.6 (40.2–42.9)	39.0 (36.9–41.2)^††^	26.9 (25.8–28.0)^††^	79.2 (77.8–80.6)^††^
**Current (past 30-day) use of e-cigarettes**					
Current nonuser (referent)					
2014	53.1 (51.9–54.4)	38.3 (37.0–39.5)	35.5 (34.3–36.8)	29.3 (28.3–30.4)	67.4 (66.3–68.6)
2015	59.0 (57.1–60.8)	40.9 (39.0–42.7)	43.8 (41.9–45.8)	29.7 (28.5–30.9)	71.9 (70.1–73.6)
2016	67.7 (66.6–68.7)	40.0 (38.8–41.2)	37.2 (35.6–38.9)	23.5 (22.5–24.6)	77.9 (76.8–78.9)
Current user					
2014	70.5 (67.3–73.6)^§§^	55.2 (52.4–57.9)^§§^	46.2 (43.6–48.8)^§§^	41.9 (38.6–45.3)^§§^	82.6 (80.4–84.7)^§§^
2015	68.4 (64.8–71.8)^§§^	56.8 (53.7–59.8)^§§^	49.1 (46.5–51.7)^§§^	41.3 (38.6–44.0)^§§^	81.8 (79.3–84.1)^§§^
2016	74.3 (70.7–77.6)^§§^	47.1 (43.4–50.8)^§§^	42.2 (39.1–45.4)^§§^	28.3 (24.8–32.0)^§§^	82.8 (79.8–85.5)^§§^
**Current (past 30-day) use, other tobacco product^¶¶^**					
Current nonuser (referent)					
2014	53.0 (51.8–54.2)	38.1 (36.8–39.5)	35.3 (34.0–36.6)	28.8 (27.7–29.9)	67.3 (66.1–68.4)
2015	59.0 (57.2–60.8)	41.2 (39.3–43.2)	43.7 (41.9–45.6)	29.7 (28.5–30.9)	72.1 (70.4–73.8)
2016	67.5 (66.4–68.6)	40.1 (39.0–41.3)	36.8 (35.2–38.5)	23.4 (22.3–24.5)	77.6 (76.6–78.6)
Current user					
2014	66.0 (63.6–68.4)^§§^	50.2 (47.5–53.0)^§§^	44.2 (42.1–46.4)^§§^	40.8 (38.3–43.3)^§§^	79.0 (77.0–80.9)^§§^
2015	66.4 (63.6–69.0)^§§^	51.8 (48.8–54.7)^§§^	49.2 (46.8–51.7)^§§^	40.0 (37.8–42.3)^§§^	78.6 (76.0–81.0)^§§^
2016	72.6 (69.4–75.6)^§§^	44.7 (41.9–47.6)^§§^	44.8 (41.6–48.0)^§§^	28.3 (25.8–30.9)^§§^	82.7 (79.7–85.4)^§§^

**Abbreviation:** CI = confidence interval.

* Exposure to each e-cigarette advertisement source was assessed by the following questions: Retail Stores: “When you go to a convenience store, super market, or gas station, how often do you see ads or promotions for e-cigarettes?”; Internet: “When you are using the internet, how often do you see ads or promotions for e-cigarettes?”; Television (TV)/Movies: In 2014, Television/movie exposure was assessed by the question “When you watch TV or go to the movies, how often do you see ads or promotions for e-cigarettes?” In 2015–2016, only TV exposures were assessed: “When you watch TV, how often do you see ads or promotions for e-cigarettes?”; and Newspaper and Magazines: “When you read newspapers or magazines, how often do you see ads or promotions for e-cigarettes?” For all questions, response options included “Never, Rarely, Sometimes, Most of the time, or Always.” A “not applicable” (N/A) response was also included to capture respondents who did not use each advertising source. Respondents were categorized as “Exposed” if they reported seeing ads or promotions “sometimes,” “most of the time,” or “always.” Respondents were categorized as “Unexposed” if they reported seeing ads or promotions “never,” or “rarely.” Individuals who reported N/A were included in the analysis in the “Unexposed” group. A composite measure of any advertisement exposure (any source) is assessed based on exposure to retail, internet, television/movies, and print ad exposures.

^†^ Population estimates rounded down to the nearest 0.1 million.

^§^ Significantly different from males at p<0.05 based on paired t-test.

^¶^ Significantly different from non-Hispanic white at p<0.05 based on paired t-test.

** Significantly different from 6th grade at p<0.05 based on paired t-test.

^††^ Significantly different from middle school at p<0.05 based on paired t-test.

^§§^ Significantly different from noncurrent users at p<0.05 based on paired t-test.

^¶¶^ Based on respondents' use of cigarettes, cigars, smokeless tobacco (includes chewing tobacco/snuff/dip, snus, and dissolvable tobacco), hookah/waterpipe, regular pipe, and/or bidis on at least one day during the past 30 days.

Overall in 2016, 28.3% of students reported exposure to e-cigarette advertising from one source, 21.2% from two sources, 16.7% from three sources, and 12.0% from four sources (Figure 2). Retail stores were the most common exposure source every year (2014: 54.8%; 2015: 59.9%; 2016: 68.0%), whereas newspapers and magazines were the least common exposure source (2014: 30.4%; 2015: 31.0%; 2016: 23.9%). The Internet was the second most common exposure source in 2014 (39.8%) and 2016 (40.6%); in 2015, television (44.5%) exceeded the Internet (42.6%) as the second most common exposure source.

**FIGURE 2:**
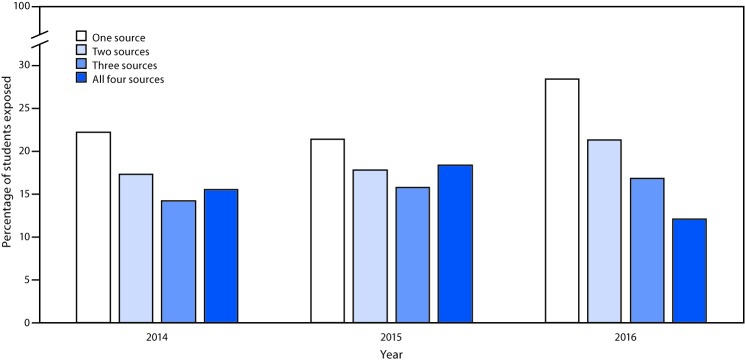
Percentage of U.S. middle and high school students who were exposed to e-cigarette advertising, by number of exposure sources*— National Youth Tobacco Survey, United States, 2014–2016 * The four exposure sources were retail stores, the Internet, television/movies, and newspapers and magazines. Movies were removed as an advertising source after 2014.

During 2014–2016, middle and high school students’ exposure to e-cigarette advertising significantly increased for retail stores (from 54.8% to 68.0%), significantly decreased for newspapers and magazines (from 30.4% to 23.9%), and did not significantly change for Internet and television.

## Discussion

In 2016, an estimated four in five (20.5 million) U.S. youths, including 8.9 million middle school students and 11.5 million high school students, were exposed to e-cigarette advertisements from at least one source, a 13% increase over 2014. Exposure in retail stores increased 24% in 2016 compared with 2014, and was the primary factor responsible for the increases in exposure from any source during 2014–2016. Nearly seven in 10 youths (17.7 million) were exposed to e-cigarette advertising in retail stores in 2016; approximately two in five were exposed on the Internet (10.6 million) or television (9.7 million), and nearly one in four (6.2 million) were exposed in newspapers and magazines. Given the Surgeon General has established that a causal relationship exists between traditional tobacco advertising and youth tobacco product initiation (*7*), and given the association between e-cigarette advertising exposure and e-cigarette use among youths (*2*–*4*), efforts to reduce youth e-cigarette advertising exposure are an important component of comprehensive youth tobacco prevention efforts (*5**)*.

During 2014–2016, current users of e-cigarettes and other tobacco products reported higher prevalence of exposure to e-cigarette advertising than nonusers. This is consistent with research documenting an association between e-cigarette advertising exposure and e-cigarette use (*2*–*4*). However, this relationship might not be limited to e-cigarettes; previous research has demonstrated that among U.S. youths aged 12–17 years, receptivity to e-cigarette marketing is associated with susceptibility to conventional cigarette smoking (*8*). Prevention of youth exposure to e-cigarette advertising might, therefore, be important for prevention of youth use of all tobacco products.

The Surgeon General has concluded that e-cigarette marketing employs strategies similar to conventional cigarette advertising tactics that have been proven to appeal to youths, such as themes of romance, freedom, and rebellion; celebrity endorsements; and health claims (*5*,*7*). Exposure to e-cigarette advertising might reduce youths’ perception of harm associated with e-cigarettes and increase their beliefs that e-cigarettes can be used where smoking is prohibited (*8*). Product design features might also influence use. For example, JUUL, the top-selling U.S. e-cigarette brand,^¶^ is an e-cigarette shaped like a USB flash drive that has a high nicotine concentration (*9*). According to news reports and social media posts, students are using JUUL in school classrooms and bathrooms (*9*).**^,^^††^ In addition, e-cigarettes are marketed and promoted using strategies that are not legally permissible for conventional cigarettes, including television, sports, and music event sponsorships, in-store self-service displays, and advertisements placed outside of brick-and-mortar businesses at children’s eye level (*5*,*10*).

As of August 2016, the Food and Drug Administration enforces restrictions on e-cigarette sales to minors, including those over the Internet.^§§^ Additional actions to reduce youths’ tobacco access and advertising exposure could include requiring that e-cigarettes are sold in adult-only facilities, limiting tobacco outlet density or proximity to schools, prohibiting self-service displays, and requiring face-to-face transactions for all e-cigarette purchases (*6*). Additional potential strategies include regulation of advertising with demonstrated youth appeal or broad youth reach at retail stores, on television, online, and in print media; and high-impact tobacco education campaigns that warn youths about the dangers of any tobacco product use, including e-cigarettes (*5*,*6*).

The findings in this study are subject to at least four limitations. First, self-reports of advertising exposure might be subject to reporting bias. Moreover, current e-cigarette users might be more likely to recall exposure than nonusers. Second, the NYTS might not be representative of all U.S. youths, because it does not capture those who are homeschooled, have dropped out of school, or are in detention centers. However, data from the Current Population Survey indicate that 98.5%, 98.0%, and 93.0% of U.S. youths aged 10–13, 14–15, and 16–17 years, respectively, were enrolled in a traditional school in 2016.^¶¶^ Third, advertising exposure might be underestimated because exposure from other potential sources such as sporting events, radio, billboards, or movies was not assessed. Finally, the removal of movies as a source of exposure after 2014 limited the comparability of television e-cigarette advertisements between years. However, this change likely resulted in an underestimation of exposure in 2015 and 2016.

Exposure to e-cigarette advertisements increased among U.S. middle and high school students during 2014–2016. As part of comprehensive youth tobacco prevention efforts, approaches to reduce youth access to e-cigarettes and exposure to e-cigarette advertising could include regulation of youth-oriented marketing, restrictions on youth access to tobacco products in retail settings, and high-impact youth-focused tobacco education campaigns (*5*). These approaches, coupled with comprehensive state tobacco control programs, have the potential to prevent and reduce youth use of all tobacco products, including e-cigarettes (*5*).

**Summary**

**What is already known about this topic?**

E-cigarettes are the most commonly used tobacco product among U.S. middle and high school students. E-cigarette advertising is associated with e-cigarette use among youths, and employs themes and strategies that are similar to conventional cigarette advertising tactics that have been proven to appeal to youths.

**What is added by this report?**

In 2016, an estimated 4 in 5 (20.5 million) U.S. middle and high school students were exposed to e-cigarette advertisements from at least one source, a significant increase over 2014 and 2015. Nearly seven in 10 youths (17.7 million) were exposed to e-cigarette advertising in retail stores in 2016, while approximately two in five were exposed on the Internet or on television, and nearly one in four were exposed through newspapers and magazines.

**What are the implications for public health practice?**

As part of comprehensive youth tobacco prevention efforts, approaches to reduce youth access to e-cigarettes and exposure to advertising could include regulation of youth-oriented marketing, restrictions on youth access to tobacco products in retail settings, and high-impact youth-focused tobacco education campaigns.

## Footnotes

* https://www.cdc.gov/tobacco/data_statistics/surveys/nyts/index.htm.

† Current use of other tobacco products is based on respondents' self-reported use of cigarettes, cigars [includes cigars, cigarillos, and little cigars], smokeless tobacco [includes chewing tobacco/snuff/dip, snus, and dissolvable tobacco], hookah/waterpipe, regular pipe, and/or bidis at least one day in the past 30 days.

§ Statistical tests for differences in e-cigarette advertisement exposure sources by year (2014, 2015, and 2016) were assessed by the Wald F-Test (ANOVA); p-values <0.05 were considered statistically significant. Posthoc comparisons for changes in e-cigarette advertisement exposures between years (2014–2015, 2015–2016, and 2014–2016) were assessed as model-adjusted risk differences from predicted marginals in logistic regression (t-test). A p-value <0.0167, adjusted for multiple comparisons, was considered statistically significant.

¶ Wells Fargo Securities, LLC. Nielsen: Tobacco ‘All Channel’ Report Ending 2.24.18.

**
https://www.reddit.com/r/juul/comments/61is7i/whats_juul_in_school/ .

††
https://www.youtube.com/watch?v=CIYQtVsOELY .

§§ https://www.fda.gov/downloads/TobaccoProducts/GuidanceComplianceRegulatoryInformation/Retail/UCM520813.pdf.

¶¶ https://www.census.gov/data/tables/2016/demo/school-enrollment/2016-cps.html.
